# Psychosocial Trauma History Negatively Impacts Liver Transplant Access in Women with Chronic Liver Disease

**DOI:** 10.1155/2024/2455942

**Published:** 2024-08-13

**Authors:** Katherine M. Cooper, Alessandro Colletta, Dhruval Amin, Darya M. Herscovici, Deepika Devuni

**Affiliations:** ^1^ Department of Medicine UMass Chan Medical School, Worcester 01655, MA, USA; ^2^ Division of Gastroenterology and Hepatology UMass Chan Medical School, Worcester 01655, MA, USA

## Abstract

**Introduction:**

Few studies have evaluated the impact of psychological trauma (mental, emotional, or physical) on liver transplant (LT) candidacy and outcomes.

**Methods:**

We performed a single center retrospective analysis of patients who completed routine LT evaluation between October 2017 and June 2021. We identified the prevalence of psychological trauma history in men and women LT candidates and evaluated the association between trauma history and LT access. The primary outcome measure was listing for LT.

**Results:**

A total of 463 patients completed LT evaluation, of which 17% (*n* = 79) reported a history of trauma: 49 of 159 women and 30 of 304 men. Trauma history was significantly more common in women than in men (31% vs. 10%, *p* < 0.001). Women with trauma history were less likely to be listed for LT (80% vs. 93%, *p* = 0.016). Women with trauma history were also more likely to be removed from the LT waitlist (26% vs. 12%, *p* = 0.045); this persists when excluding patients removed for transfer to another center or for medical improvement (22% vs. 7%, *p* = 0.020). In contrast, listing for LT (87% vs. 86%, *p* = 0.973) and waitlist removal (12% vs. 10%, *p* = 0.766) did not differ in men with and without trauma history. In those that received a LT (*n* = 107), post-LT relapse, rejection, readmissions, and death did not differ in patients with (n=13)and without (n=94) trauma history.

**Conclusions:**

Trauma history is associated with reduced access to LT in women but not men with chronic liver disease. Further studies are needed to understand the impact of psychological trauma on LT access and post-LT outcomes.

## 1. Introduction

Up to 90% of adults in the United States experience at least one potentially traumatic event (PTE) in their lifetime, but the prevalence of post traumatic stress disorder (PTSD) is less than 10% [1]. Instead, many patients experience distressing symptoms that can lead to developing non-PTSD psychiatric comorbidities such as mood and substance-use disorders after a PTE [2]. Psychological trauma is a broad term that encompasses any emotional, painful, distressful, or shocking experiences that result in long term mental and physical effects [2]. Rather than a criteria-based diagnosis, psychological trauma relies on an individual's subjective experience [1]. Because individuals perceive PTEs differently, behavioral response, emotional response, and long-term impact can vary from patient to patient [2].

There is a wide breadth of literature on sex and gender-based differences in PTE exposure and response. Though there is considerable geographic and cultural variation in this, women are more likely to experience sexual and partner violence and men are more likely to experience combat-related trauma or trauma related to witnessing the harm of others [3–5]. Women are at greater risk of developing comorbid psychiatric conditions after a PTE, including a twofold greater risk of PTSD when compared to men [4]. Some data suggest that men are more like to develop substance use disorders, though substance use is common in women with trauma history as well [5]. There is significant interplay between trauma and the risk of developing an alcohol use disorder, where either of these may increase the risk of developing the other [6].

The prevalence of trauma-related disorders in patients with cirrhosis is not widely reported, though the prevalence of PTSD may be as high as 34% in patients with severe alcohol use disorder who have developed liver disease [7]. When present, there is potential for psychological trauma to impact liver transplant (LT) outcomes. Trauma history has been associated with alcohol and substance relapse [8–10] and poor medication adherence in patients with liver disease [9, 11, 12]. In a very small study of pediatric LT recipients, childhood trauma was associated with a higher risk of transplant rejection [13, 14]. Whether trauma history is routinely assessed during adult LT evaluation is not clear, as it is not specifically addressed in the literature related to the psychosocial evaluation [15, 16]. As such, the influence of psychological trauma (mental, emotional, or physical) on adult LT is not well described. Our primary objective was to assess for association between psychological trauma history and access to LT in patients with advanced liver disease, with a focus on gender differences.

## 2. Methods

### 2.1. Patient Selection

We performed a single center retrospective analysis of patients with cirrhosis who underwent routine LT evaluation at a large academic tertiary care and LT center between October 2017 and June 2021. Patients undergoing inpatient LT evaluation were excluded, as there are several factors that may interfere with collecting an accurate psychiatric and psychosocial history in the inpatient setting (e.g., hepatic encephalopathy, respiratory failure requiring intubation, and use of collateral information or interviewing family members).

### 2.2. Defining Trauma History

Each patient underwent a complete psychosocial and psychiatric assessment conducted by experienced transplant psychiatrists and transplant social workers on the multidisciplinary transplant team. Evaluations were performed in clinic or via telehealth during the COVID-19 pandemic and support persons were present during the evaluation at the discretion of the patient. All patients were informed of the role of the transplant social worker and psychiatrists and the intended purpose and documentation of information collected during the interview. During the interview, patients were asked if they had a history of trauma in the form of an open-ended question. Because the experience of trauma is subjective, patients were categorized into one of two groups, “trauma” or “no trauma,” based on their response to this question. Patients reporting trauma related to the liver disease diagnosis or the transplant process were not included in the trauma group. Those with a history of trauma were further characterized by the source and type of trauma. Category selection was informed by existing psychology literature and trends identified in our patient cohort [5]. The source of trauma was categorized as one of the following: childhood, adult, work related, death of another person, and others. The type of trauma was categorized as one of the following: sexual violence, physical violence, experienced event without direct harm, physical trauma without directed violence, and other. Examples of trauma type can be found in Supplemental Table 1.

### 2.3. Data Collection

Data were extracted directly from the electronic health record. Demographic data included age, race (White vs. non-White), ethnicity (Hispanic vs. non-Hispanic), highest level of education (high-school vs. >high school), and primary language (English vs. non-English). Biologic sex and patient gender were collected; because all patients were cisgender, male and female patients are referred to as men and women, respectively. Liver disease etiology was defined using the primary diagnosis in the transplant encounter; primary biliary cholangitis, primary sclerosing cholangitis, and autoimmune hepatitis were collapsed to autoimmune liver diseases (AILDs) for analytic simplicity. Body mass index (BMI) and Model for End Stage Disease-Sodium (MELD-Na) score were collected from the time of LT evaluation. Psychosocial variables included relationship status (partner vs. no partner), occupational status in the preceding year (employed vs. unemployed), and living situation (lives with family vs. does not live with family). Comorbid psychiatric disorders (anxiety, depression, and bipolar depression) and substance-use history (tobacco, cocaine, marijuana, and opioid) were collected as well. Post-LT substance use, biopsy-proven rejection, hospital readmissions, and two-year survival were recorded for patients who underwent LT. Data were managed using the REDCap electronic data capture tools at our institution [17, 18].

### 2.4. Analysis

The primary aim of the analysis was to assess for association between psychological trauma history and LT access in women and men. Outcome measures included waitlist registration (listing), waitlist removal, and receipt of LT. Multivariable logistic regression was used to evaluate for association between trauma history and outcomes measures. We opted to use augmented backward logistic regression for these analyses to increase the reliability of the model by accounting for pertinent demographic and psychosocial factors [19, 20]. The following variables were included on initial entry: trauma history, age, MELD-Na, BMI, race, ethnicity, liver disease etiology, education, employment, primary language, relationship status, living with family, depression, anxiety, psychotropic medications, tobacco use, cocaine use, marijuana use, and opioid use. Strength of association is reported as odds ratio (OR) with 95% confidence interval (95% CI). Statistical analyses were performed in SPSS 29. This study was reviewed and approved with a waiver of informed consent by the institutional review board at our medical center.

## 3. Results

### 3.1. Patient Characteristics

A total of 463 patients completed routine LT evaluation, of which 17% reported a history of trauma (49 of 159 women and 30 of 304 men). Patients with trauma history were younger (*p* < 0.001) and more likely to be White (*p* = 0.032). Women were significantly more likely to report a history of trauma than men (31% vs. 10%, *p* < 0.001) (Table 1). In general, patients were diagnosed with liver disease 3–4 years prior to LT evaluation. Mean BMI (*p* = 0.229) and MELD-Na scores (*p* = 0.676) were similar between the groups (Table 1).

There were multiple psychosocial differences in patients with and without trauma history. Patients with a history of trauma were less likely to be living with a family member (58% vs. 76%, *p* = 0.002) and less likely to have a stable relationship partner (42% vs. 63%, *p* < 0.001) (Table 1). Psychiatric comorbidities including depression (*p* < 0.001), anxiety (*p* < 0.001), and bipolar disorder (*p* < 0.001) were more prevalent in patients with a history of trauma, and these patients were more likely to be prescribed a psychotropic medication at the time of LT evaluation (51% vs. 26%, *p* < 0.001) (Table 1). Prior diagnosis of acute alcohol associated hepatitis (AAH) was significantly more common in patients with a history of trauma (32% vs. 14%, *p* < 0.001). Substance-use history including marijuana (*p* = 0.028), opioid (*p* = 0.002), and cocaine (*p* < 0.001) use were more common as well, though tobacco-use history did not differ between the groups (*p* = 0.136) (Table 1). The relationship between trauma history andseveral psychosocial variables observed in the total cohort differed when stratified by gender (Table 1).

#### 3.1.1. Trauma History

Of the patients that reported a history of trauma (*n* = 79), 87% patients completed their psychosocial evaluation in clinic and 67% were accompanied by a support person. Almost all patients reported the source of their trauma history (*n* = 76, 96%). Childhood trauma was the most common source of trauma history (*n* = 31, 39%) followed by physical abuse and/or intimate partner violence (*n* = 18, 23%). Most patients also shared details of their trauma history (*n* = 66, 86%). Twenty-seven patients (34%) reported experiencing physical violence and twelve patients (15%) reported trauma related to sexual violence. Patients with a history of sexual trauma were 5.3 times more likely to be a woman (95% CI 1.1–25.6, *p* = 0.040), and trauma related to partner violence was exclusively reported in women (18 of 49 women). Disclosure of trauma details did not differ in patients who completed their evaluation in clinic compared to via telehealth (86% vs. 90%, *p* = 0.701) or in patients who were accompanied during their visit compared to alone (85% vs. 87%, *p* = 0.668). A similar proportion of men and women provided details of their trauma history (80% vs. 90%, *p* = 0.222). See Table 2 for a summary of trauma experiences and Supplemental Table 1 for examples within each categorization.

### 3.2. Study Outcomes

**Figure 1:**
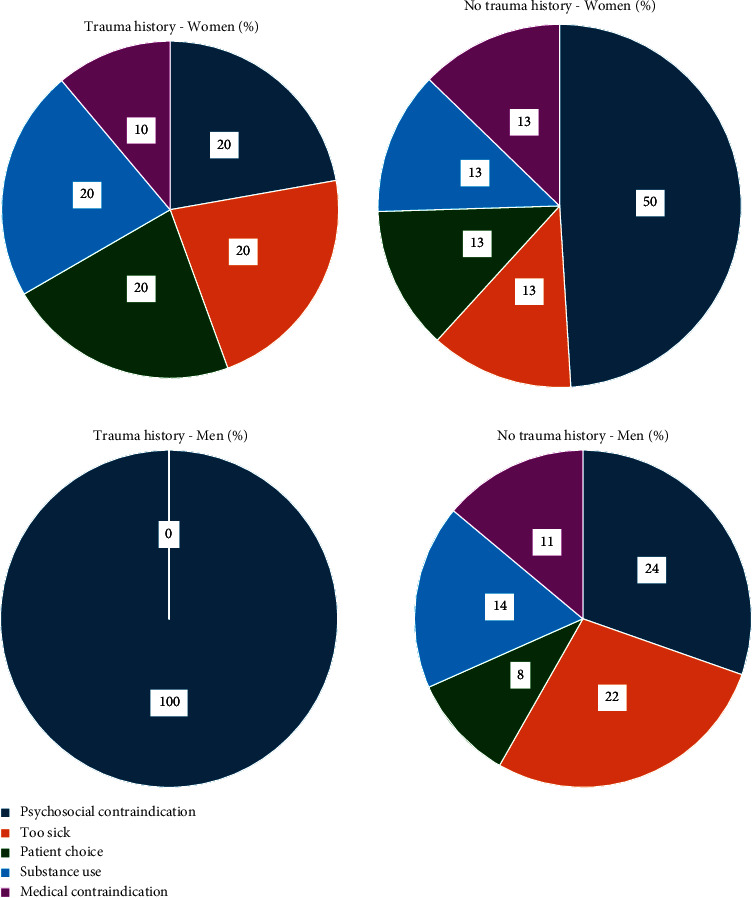
Reasons patients were declined for listing by patient gender and trauma history group.

In total, 14 of 79 (18%) of patients with a history of trauma and 45 of 384 (12%) patients without a history of trauma were declined for listing during LT evaluation. The most common reasons patients were declined for listing were clinical improvement (*n* = 12, 20%), medical contraindication to transplant (*n* = 12, 20%), and substance use (*n* = 12, 20%). Of those declined, patients with trauma history were more likely to be declined for substance use (43% vs. 13%, *p* = 0.017) (Figure 1).

#### 3.2.1. Waitlist Access

**Figure 2:**
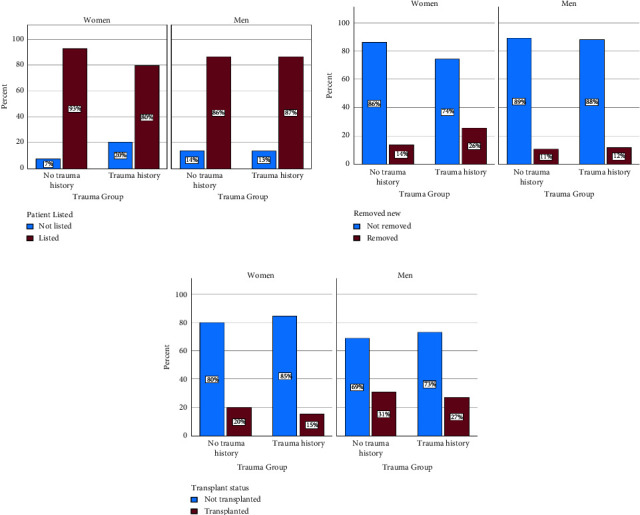
Transplant-related outcomes by patient gender and trauma history, reported as percentages. (a) Listing rates by patient gender and trauma history group. Trauma history was associated with lower waitlist registration in women (*p*=0.016) but not in men (*p*=0.973). (b) Waitlist removal by patient gender and trauma history group. Trauma history was associated with higher waitlist removal in women (*p*=0.027) but not in men (*p*=0.761). (c) Transplant rate by patient gender and trauma history group. Fewer men and women with trauma history received a liver transplant, though this did not reach statistical significance (*p*=0.121).

Women with trauma history were significantly less likely to be listed for LT than women without trauma history (80% vs. 93%, *p* = 0.016) (Figure 2(a)). The reasons for which women were declined for listing did not differ between those with and without trauma history. On multivariable analysis, trauma history was independently associated with being declined for listing in women (OR 0.2, 95% CI 0.1–0.69, *p* = 0.011, Table 3). Waitlist enrollment did not differ in men with and without a history of trauma (87% vs. 86%, *p* = 0.973) (Figure 2(a)).

#### 3.2.2. Waitlist Removal

Of those that were listed for LT, 10 of 39 (26%) women with trauma history were removed from the waitlist compared to 14 of 102 (14%) women without trauma history (Figure 2(b)). Excluding those removed due to improvement in condition (*n* = 4), at patient request (*n* = 3), or at the time of transfer to another center (*n* = 2), women with trauma history were significantly more likely to be removed from the waitlist compared to women without trauma history (22% vs. 7%, *p* = 0.020). Women with trauma history were more likely to be removed for substance use or poor psychosocial support than women without trauma history (70% vs. 17%, *p* = 0.027). On multivariable analyses, trauma history was associated with over fivefold greater odds of being removed from the LT waitlist for unfavorable reasons (OR 5.8, 95% CI 1.6–20.8, *p* = 0.013) (Table 4). The rate of waitlist removal did not differ by trauma group in men (12% vs. 10% *p* = 0.761) (Figure 2(b)).

#### 3.2.3. Transplant Outcomes

A total of 13 patients with a history of trauma (6 women and 7 men) and 94 patients without a history of trauma (20 women and 74 men) underwent LT (Figure 2(c)). Fewer patients with trauma history underwent LT than patients without trauma history (17% vs. 25% *p* = 0.121). The mean MELD-Na score at the time of LT did not differ in patients with and without trauma history (21 vs. 19, *p* = 0.613). The mean number of hospital readmissions within one year of LT was similar in patients with and without pre-LT trauma history (*p* = 0.513) and about one half had 2 or more readmissions (54% trauma vs. 48% no trauma, *p* = 0.682). The rate of biopsy proven acute cellular rejection did not differ based on pre-LT trauma history (8% trauma vs. 24% no trauma, *p* = 0.198). Alcohol relapse was similar between groups as well. Of those with ALD, 1 of 9 patients with a pre-LT trauma history experienced alcohol relapse within two years of LT compared to 6 of 54 patients without pre-LT trauma history (*p* = 1.000). Post-LT outcomes were not analyzed by gender due to the sample size. See Table 5 for a summary of post-LT metrics.

## 4. Discussion

Our results suggest that a history of psychological trauma is associated with reduced access to LT, particularly among women. One in three LT candidates in our study was a woman, which is consistent with known trends of liver disease in the United States [21, 22]. Women were more likely to report a history of trauma than men. Women with a history of trauma were less likely to be listed for LT and more likely to be removed from the LT waitlist compared to women without a history of trauma. In contrast, there were no differences in listing or waitlist removal in men with and without a history of trauma. Importantly, in patients who received a LT, trauma history did not affect post-LT rejection, substance relapse, or readmission rates.

We analyzed a considerable number of variables to determine potential drivers of the observed gender differences. Trauma history has been associated with mood and substance use disorders [23]. Women with a history of trauma had the highest prevalence of ALD in our study, but high risk alcohol use did not appear to differ between genders. Approximately 30–40% of both men and women with a history of trauma had a prior diagnosis of AAH compared to 15% of both men and women without a history of trauma. This suggests that trauma history had a similar association with high-risk drinking behaviors in both genders.

With respect to substance use, women with trauma history were less likely to report prior substance use than men. Furthermore, 100% of the men with a history of trauma that were declined for listing were declined for substance use compared to 20% of women with a history of trauma. This finding may be related to higher rates of cocaine use and hepatitis C virus (HCV) in men in our study. While the trend of increased substance use in men with trauma history was consistent with prior studies, it is somewhat counterintuitive as trauma history did not affect LT access in men.

We did observe higher rates of anxiety and depression in women than men, and previous studies have shown that women are more likely to develop anxious and depressive disorders after a PTE. However, none of the patients in the study were declined for listing or removed from the waitlist for poorly controlled psychiatric comorbidities. Furthermore, we did not find depression to be associated with listing on our multivariable analysis. These findings suggest that mood and substance use disorders are not the drivers of the gender disparity observed in this study.

Different levels of psychosocial support may play a role in our findings of reduced LT access in patients with trauma history. Trauma history is associated with long-term effects on interpersonal connections and relationships [2, 24]. Both men and women with trauma history were less likely to live with family members and less likely to have a stable relationship at the time of LT evaluation. However, women with trauma history were more likely to be declined for listing due to psychosocial reasons compared to both men with trauma and women without trauma. Women with trauma history were also more likely to be lost to follow up during the evaluation process, which may reflect lower levels of support. The connection between gender and psychosocial support may be influenced by the type of trauma experienced. Specifically, sexual trauma has been associated with greater impairments in interpersonal relationships [25], and women in our study were significantly more likely to experience sexual trauma. Though we were unable to account for this in our study, it is possible that reduced psychosocial support in women with trauma history was a stronger driver of the observed gender differences than substance use or mood disorders.

Limitations of this study include its single center and retrospective design. While these factors may affect the generalizability of our findings, the study's sample size and conduct at a safety net hospital might mitigate some of these limitations. Our study also relied on self-reported trauma history and may be subject to recall or reporting bias. To minimize these biases, we focused our analysis on patients evaluated in the outpatient setting, where they were neither acutely ill nor affected by overt encephalopathy. Our study had a limited racial representation with a notably lower proportion of Black and African American patients than expected compared to the national LT population. While this is representative of the population in Massachusetts, we were not able to address potential differences related to race. In addition, it should be noted that a relatively small number of patients with a history of trauma underwent LT and findings related to post-LT outcomes should be interpreted with caution. Despite these limitations, we believe these results are important due to the profound association between psychological trauma history and reduced access to LT. All patients at our institution were asked about trauma history, but it is not known whether trauma history is routinely addressed during the LT evaluation at other institutions. Currently, assessment of trauma history is not discussed in practice guidance about the psychosocial evaluation and is not included in well-known psychosocial evaluation tools like the SIPAT [15, 16, 26]. These factors in conjunction with the lack of published data on this topic suggest that trauma history many not be routinely queried during the LT evaluation process. Bringing attention to this may be of increasing importance in the post-COVID-19 era given the number of young people reporting trauma related to the pandemic [27]. Future research should investigate the association between trauma history and post-LT outcomes, and explore the relationship between psychological trauma and access to transplantation in other solid organ transplants. Futhermore, qualitative studies assessing for system or provider level bias towards women with complex psychosocial history are needed to further address this disparity.

## Figures and Tables

**Table 1:** Patient demographics by Trauma and No trauma status in men, women, and the total cohort.

	All patients			Women			Men		
	No trauma	Trauma	*p*	No trauma	Trauma	*p*	No trauma	Trauma	*p*
Age (years)	58 (10)	52 (10)	<0.01	57 (10)	52 (10)	0.01	58 (11)	51 (10)	<0.01
BMI (kg/m^2^)	30 (7)	29 (7)	0.23	29 (7)	28 (7)	0.46	30 (6)	30 (7)	0.93
MELD-Na (points)	13 (6)	13 (7)	0.68	13 (6)	13 (6)	0.84	13 (6)	13 (7)	0.70
Gender (% women)	28.7	62.0	<0.01	−	−	−	−	−	−
Hispanic (%)	12.0	8.5	0.37	13.4	4.2	0.08	11.5	14.7	0.58
Race (%)									
White race	83.0	91.5	0.03	82.7	93.9	0.06	83.0	90.0	0.32
Black	3.4	0.0	−	4.5	0.0	−	2.9	0.0	−
Asian	2.3	2.5	0.92	2.7	2.0	0.80	2.2	3.3	0.69
Other	11.5	5.1	0.09	10.0	4.1	0.31	12.0	6.7	0.38
Etiology (%)									
ALD	47.3	57.0	0.12	37.3	61.2	<0.01	51.3	50.0	0.89
MASLD	25.6	12.7	0.01	30.0	12.2	0.02	23.8	13.3	0.19
HCV	14.6	19.0	0.33	9.1	14.3	0.33	16.8	26.7	0.18
AILD	6.3	5.1	0.68	17.3	8.2	0.13	1.8	0.0	−
Other	6.3	6.3	0.98	6.4	4.1	0.57	6.2	10.0	0.43
Psychosocial (%)									
>High school education	49.5	51.9	0.69	54.7	51.1	0.68	47.3	53.3	0.53
Employed in last year	45.0	35.8	0.13	39.1	34.0	0.55	47.4	38.2	0.31
Military history	13.5	14.6	0.79	2.7	4.2	0.63	18.0	29.4	0.11
Lives with family	76.3	56.1	<0.01	76.6	56.3	0.01	76.2	55.9	0.01
Stable pPartner	62.9	41.5	<0.01	55.0	41.7	0.12	66.2	41.2	<0.01
Routine outpatient care	86.6	84.1	0.55	86.6	82.5	0.59	87.0	87.0	1.00
English as 2^nd^ language	11.5	10.1	0.72	11.9	6.1	0.26	11.4	16.7	0.40
Substance-use history (%)									
AAH	14.1	31.6	<0.01	11.8	28.6	<0.01	15.0	36.7	<0.01
Opioid	13.1	23.8	0.02	11.2	25.5	0.02	13.8	21.2	0.26
Marijuana	16.8	31.7	0.02	9.8	33.3	<0.01	19.8	29.4	0.19
Cocaine	12.9	31.7	<0.01	6.3	25.0	<0.01	15.7	41.2	<0.01
Tobacco	58.7	70.4	<0.01	56.2	77.2	0.02	59.7	60.6	0.02
Comorbid psychiatric disorders (%)									
Depression	32.8	64.6	<0.01	43.8	72.9	<0.01	28.3	52.9	<0.01
Anxiety	23.3	43.9	<0.01	27.7	52.1	<0.01	21.5	32.4	0.15
Bipolar disorder	2.4	7.3	0.02	4.5	8.3	0.33	1.5	5.9	0.08
Post-LT outcomes (%)									
≥2 hospital admissions	47.8	53.8	0.68	−	−	−	−	−	−
Biopsy-proven rejection	23.7	7.7	0.19	−	−	−	−	−	−
Alcohol relapse	11.1	11.1	1.00	−	−	−	−	−	−
2-year survival	79.3	100	0.20	−	−	−	−	−	−

Post-LT outcomes were evaluated in the total cohort only due to sample size. Demographics are compared between patients with (+) and without (−) a history of trauma in the total cohort, in men, and in women using chi squared or Students *t* tests. Significance was evaluated at *p* < 0.05. BMI: body mass index; MELD-Na: Model for End Stage Liver Disease-Sodium score; ALD: alcohol-associated liver disease; MASLD: metabolic dysfunction-associated steatotic liver disease; HCV: chronic hepatitis C; AILD: autoimmune liver diseases.

**Table 2:** Trauma experiences by patient gender.

	Women (%)	Men (%)	Total (%)	*p* values
*Source of trauma*				
Childhood (physical, sexual)	32.7	50.0	39.2	0.125
Adult (physical, sexual IPV)	36.7	0.0	22.8	—
Work related	4.1	20.0	10.1	0.023
Death of another person	10.2	16.7	12.7	0.402
Other personal event	12.2	10.0	11.4	0.761
No details provided	4.1	3.3	3.8	0.866

*Trauma details*				
Sexual violence	22.4	3.3	15.2	0.022
Physical violence	30.6	40.0	34.2	0.393
Experienced event without physical harm	26.5	40.0	31.6	0.212
Physical trauma without directed violence	6.1	3.3	5.1	0.583
Not enough detail	14.3	13.3	13.9	0.906

**Table 3:** Predictors of listing for LT among women LT candidates using multivariable and backward logistic regression analyses.

Variable	aOR	CI	*p*	bOR	CI	*p*
Trauma	0.19	0.04–0.92	0.04	0.20	0.06–0.68	0.01
Age	0.96	0.88–1.05	0.41	—	—	—
MELD-Na	0.94	0.84–1.06	0.32	—	—	—
BMI	1.15	1.00–1.33	0.05	1.14	1.03–1.28	0.02
Race [White]	0.37	0.02–6.14	0.49	—	—	—
Ethnicity [Hispanic]	4.95	0.26–92.7	0.29	—	—	—
Etiology [ALD]	—	—	0.33	—	—	0.09
MASLD	0.14	0.02–1.17	0.07	0.17	0.03–0.88	0.04
HCV	0.19	0.02–1.90	0.16	0.14	0.03–0.75	0.02
AILD	1.66	0.11–24.6	0.71	1.15	0.12-11.4	0.90
Other	0.27	0.01–4.94	0.38	0.16	0.01–2.08	0.16
Education [≤HS]	2.84	0.58–13.8	0.20	—	—	—
Employed	2.44	0.52–11.3	0.26	—	—	—
Language	0.61	0.04–10.6	0.73	—	—	—
Partner	0.68	0.11–4.12	0.68	—	—	—
Lives with family	0.70	0.13–3.85	0.68	—	—	—
Depression	1.03	0.13–8.49	0.98	—	—	—
Anxiety	0.67	0.12–3.70	0.65	—	—	—
Psychotropic medications	0.53	0.09–3.27	0.49	—	—	—
Tobacco	2.24	0.43–11.7	0.34	—	—	—
Cocaine	0.73	0.08–6.60	0.78	—	—	—
Marijuana	0.52	0.07–3.63	0.51	—	—	—
Opioid	1.75	0.22–14.0	0.60	—	—	—

A total of 20 additional clinically relevant variables, including depression and anxiety, were entered into a single-entry multivariable model (left) to evaluate whether trauma history was associated with listing for LT. Backward conditional logistic regression was applied to identify independent predictors of being listed (right). Trauma history was associated with lower odds of listing and was identified as an independent predictor of the listing outcome. Additional predictors include disease etiology and BMI. LT: liver transplant; BMI: body mass index; MELD-Na: model for end stage liver disease-sodium score; ALD: alcohol-associated liver disease; MASLD: metabolic dysfunction-associated steatotic liver disease; HCV: chronic hepatitis C; AILD: autoimmune liver diseases.

**Table 4:** Multivariable and backward logistic regression for predictors of being removed from the LT waitlist in women.

Variable	aOR	CI	*p*	bOR	CI	*p*
Trauma	5.52	0.9–35.9	0.10	5.80	1.6–20.8	0.01
Age	1.00	0.9–1.1	0.97	—	—	—
MELD-Na	1.15	1.0–1.3	0.02	1.10	1.0–1.2	0.05
BMI	1.09	1.0–1.3	0.08	—	—	—
Etiology [ALD]	—		—	—	—	—
MASLD	0.13	0.0–2.8	0.40	—	—	—
HCV	0.17	0.0–2.7	0.28	—	—	—
Other	0.17	0.0–2.5	0.13	—	—	—
Education [≤HS]	1.06	0.2–5.7	0.41	—	—	—
Employed	0.39	0.1–2.4	0.05	—	—	—
Partner	6.19	1.0–40.3	0.03	3.28	0.9–12.6	0.08
Depression	0.52	0.0–6.2	0.31	—	—	—
Anxiety	0.20	0.0–1.7	0.40	—	—	—
Psychotropic medications	6.89	0.6–81.2	0.12	—	—	—
Tobacco	1.32	0.2–9.3	0.68	—	—	—
Cocaine	0.15	0.0–1.8	0.27	—	—	—
Marijuana	5.46	0.9–35.1	0.35	—	—	—
Opioid	0.57	0.1–4.2	0.87	—	—	—

A total of 20 additional clinically relevant variables including depression and anxiety were entered into a single entry multivariable model (left) to evaluate for association between a history of trauma history and being removed from the LT waitlist. Backward conditional logistic regression was applied to identify independent predictors of being waitlisted. BMI: body mass index; MELD-Na: Model for End Stage Liver Disease-Sodium score; ALD: alcohol-associated liver disease; MASLD: metabolic dysfunction-associated steatotic liver disease; HCV: chronic hepatitis C; AILD: autoimmune liver diseases.

**Table 5:** Post-LT metrics in patients with and without pre-LT trauma history.

	No trauma history (*n* = 94)	Trauma history (*n* = 13)	*p*
MELD-Na at LT	19.4 (10)	20.8 (8)	0.613
Hospital admissions (mean #)	2 (2)	3 (2)	0.513
≥2 hospital admissions (%)	47.8	53.8	0.682
Biopsy-proven rejection (%)	23.7	7.7	0.191
Alcohol relapse (%)	11.1	11.1	1.000
2-year survival (%)	79.3	100	0.200

For alcohol relapse, only patients with pre-LT alcohol use were included in calculations (*n* = 9 trauma, *n* = 54 no trauma). For biopsy-proven rejection, all cases were acute cellular rejection within two years of LT.

## Data Availability

The data used to support the findings of this study may be available on request from the corresponding author.

## Abbreviations

AILD: Autoimmune liver diseases
PTSD: Posttraumatic stress disorder
LT: Liver transplant
MELD-Na: Model for End Stage Disease-Sodium
BMI: Body mass index
DSM: Diagnostic and statistical manual of mental disorder
OR: Odds ratio
95% CI: 95% confidence interval
ALD: Alcohol-associated liver disease
MASLD: Metabolic dysfunction-associated steatotic liver disease
HCV: Chronic hepatitis C
AAH: Acute alcohol-associated hepatitis.

## Supplementary Materials

Supplementary Materials Supplementary Table 1: specific examples of traumatic experiences reported by patients in the study grouped by trauma subcategories used in the text.

## References

[1] Kilpatrick D. G., Resnick H. S., Milanak M. E., Miller M. W., Keyes K. M., Friedman M. J. (2013). National estimates of exposure to traumatic events and PTSD prevalence using DSM-IV and DSM-5 criteria. *Journal of Traumatic Stress*.

[2] Protocol A. T. I. (2014). *Trauma-informed Care in Behavioral Health Services*.

[3] Olff M. (2017). Sex and gender differences in post-traumatic stress disorder: an update. *European Journal of Psychotraumatology*.

[4] Vogt D. (2007). Research on women, trauma and PTSD. *National Center for PTSD*.

[5] Tolin D. F., Foa E. B. (2006). Sex differences in trauma and posttraumatic stress disorder: a quantitative review of 25 years of research. *Psychological Bulletin*.

[6] Smith N. D. L., Cottler L. B. (2018). The epidemiology of post-traumatic stress disorder and alcohol use disorder. *Alcohol Res*.

[7] Samala N., Lourens S. G., Shah V. H. (2018). Posttraumatic stress disorder in patients with heavy alcohol consumption and alcoholic hepatitis. *Alcoholism: Clinical and Experimental Research*.

[8] Neuberger J. (2020). Liver transplantation for alcoholic liver disease: what is the risk and consequence of relapse?. *Digestive Diseases and Sciences*.

[9] Annema C., Drent G., Roodbol P. F. (2017). A prospective cohort study on posttraumatic stress disorder in liver transplantation recipients before and after transplantation: prevalence, symptom occurrence, and intrusive memories. *Journal of Psychosomatic Research*.

[10] Dong M., Dube S. R., Felitti V. J., Giles W. H., Anda R. F. (2003). Adverse childhood experiences and self-reported liver disease: new insights into the causal pathway. *Archives of Internal Medicine*.

[11] Douglas K. R., Chan G., Gelernter J. (2010). Adverse childhood events as risk factors for substance dependence: partial mediation by mood and anxiety disorders. *Addictive Behaviors*.

[12] Brown S. M., Shillington A. M. (2017). Childhood adversity and the risk of substance use and delinquency: the role of protective adult relationships. *Child Abuse & Neglect*.

[13] Shemesh E., Annunziato R. A., Yehuda R. (2007). Childhood abuse, nonadherence, and medical outcome in pediatric liver transplant recipients. *Journal of the American Academy of Child & Adolescent Psychiatry*.

[14] Duncan-Park S., Danziger-Isakov L., Armstrong B. (2022). Posttraumatic stress and medication adherence in pediatric transplant recipients. *American Journal of Transplantation*.

[15] Winder G. S., Fernandez A. C., Perumalswami P. V., Mellinger J. L., Clifton E. G. (2024). Reexamining “psychosocial clearance”: a procedural framework for psychosocial evaluation in liver transplantation. *Liver Transplantation*.

[16] Martin P., DiMartini A., Feng S., Brown R. J., Fallon M. (2014). Evaluation for liver transplantation in adults: 2013 practice guideline by the American association for the study of liver diseases and the American society of transplantation. *Hepatology*.

[17] Harris P. A., Taylor R., Thielke R., Payne J., Gonzalez N., Conde J. G. (2009). Research electronic data capture (REDCap)—a metadata-driven methodology and workflow process for providing translational research informatics support. *Journal of Biomedical Informatics*.

[18] Harris P. A., Taylor R., Minor B. L. (2019). The REDCap consortium: Building an international community of software platform partners. *Journal of Biomedical Informatics*.

[19] Heinze G., Dunkler D. (2017). Five myths about variable selection. *Transplant International*.

[20] Bursac Z., Gauss C. H., Williams D. K., Hosmer D. W. (2008). Purposeful selection of variables in logistic regression. *Source Code for Biology and Medicine*.

[21] Abboud Y., Mathew A. G., Meybodi M. A. (2024). Chronic liver disease and cirrhosis mortality rates are disproportionately increasing in younger women in the United States between 2000-2020. *Clinical Gastroenterology and Hepatology*.

[22] Sarkar M., Watt K. D., Terrault N., Berenguer M. (2015). Outcomes in liver transplantation: does sex matter?. *Journal of Hepatology*.

[23] Khoury L., Tang Y. L., Bradley B., Cubells J. F., Ressler K. J. (2010). Substance use, childhood traumatic experience, and Posttraumatic Stress Disorder in an urban civilian population. *Depression and Anxiety*.

[24] Goldstein E., King C., Crits‐Christoph P., Connolly Gibbons M. B. (2023). The association between trauma and interpersonal problems in a United States community mental health setting. *Journal of Clinical Psychology*.

[25] Huh H. J., Kim S. Y., Yu J. J., Chae J. H. (2014). Childhood trauma and adult interpersonal relationship problems in patients with depression and anxiety disorders. *Annals of General Psychiatry*.

[26] Maldonado J. R., Dubois H. C., David E. E. (2012). The stanford integrated psychosocial assessment for transplantation (SIPAT): a new tool for the psychosocial evaluation of pre-transplant candidates. *Psychosomatics*.

[27] Ionio C., Ciuffo G., Villa F., Landoni M., Sacchi M., Rizzi D. (2022). Adolescents in the covid net: what impact on their mental health?. *Journal of Child & Adolescent Trauma*.

