# Supplementary material for: Psychosocial Trauma History Negatively Impacts Liver Transplant Access in Women with Chronic Liver Disease
Source: J Transplant. 2024 Aug 13;2024:2455942. doi: 10.1155/2024/2455942 (PMC11335414; doi:10.1155/2024/2455942)
Supplement: Supplementary Materials — Supplementary Table 1: specific examples of traumatic experiences reported by patients in the study grouped by trauma subcategories used in the text. [file 2455942.f1.docx]

Supplemental Table 1

| **Examples** | |
| --- | --- |
| **Child** | - Sexual abuse - Physical abuse - Severe physical punishment |
| **Adult** | - Physical assault - Sexual assault - Emotional abuse |
| **Work related** | - Witnessed death as first-responder - Combat related trauma |
| **Death of another person** | - Child was murdered - Discovered person who committed suicide - Child died when 1 year old |
| **Other personal event** | - Stuck in a house fire - Serious car accident with medical trauma - Mistreatment in prison |
| **Experienced event without physical harm** | - Witnessed death as first-responder - Discovered person who committed suicide - Emotional abuse during relationship |
| **Physical trauma without directed violence** | - Car accident - Stuck in a house fire |
